# Supplementary material for: Enhancement of visual biological motion recognition in early-deaf adults: Functional and behavioral correlates
Source: PLoS One. 2020 Aug 10;15(8):e0236800. doi: 10.1371/journal.pone.0236800 (PMC7416928; doi:10.1371/journal.pone.0236800)
Supplement: Attachment — Submitted filename: Response to Reviewers.docx [file pone.0236800.s001.docx]

We are happy to enclose a revised version of the manuscript PONE-D-19-34925 entitled “Enhancement of Visual Biological Motion Recognition in Early-Deaf Adults: Functional and Behavioral Correlates” for consideration in *Plos One*.

We have carefully taken all the reviewer's suggestions into consideration to prepare a revised version of the manuscript, which also required modifications in the text and a revised figure. We truly feel the reviewers have contributed to improving the quality and clarity of the manuscript. Therefore, we would like to thank both reviewers for their precise and useful comments and suggestions as well as for their time spent reviewing our work.

Below, you can find our responses to the comments of Reviewers 1 and 2 as well as actions taken in the the revised manuscript.

We hope that you will find our revised manuscript satisfactory for publication and would be very proud to be given the opportunity to represent your journal.

**Report of Reviewer #1**

The work contrasts the recognition of different inferred movements on deaf and hearing population. The study aims to study is rooted not only in exploring cortical processing related to these movements and population characteristics, but also to link this cortical processing with behavioral differences.
In general, the article concludes that minor behavioral differences are found, which are weakly related to STG activity. Cortical activity presents evident differences between groups and conditions.
This is a well written article with a robust experiment design. Methodologically speaking it presents a classic approach properly applied.

However, there are still some room for improvement. The article may improve a few things to facilitate reader’s comprehension, and in my personal opinion, this article may use some different methodological approaches to improve the results they based on in part of the discussion. I think that authors have better results than what they think, but the technique used, does not allow them to catch such results entirely. Finally, there are some methodological decisions that are not argued, and they should be. Main reasons are to improve the understanding of the logic behind those calls, but also to improves readers’ experience and allow people who are naïve to statistics to understand the justifications of the method used.

Specifically:
1. ***Some results are given in methods sections, which I don’t clearly see why aren’t they in results section.***

**Response**: Thank you for your pertinent comment. As you mentioned, some results were given in the method section. Thus, we removed the following sentence: “Deaf and hearing groups were equivalent with regards to age (t(30) = .035, p = .682), number of years of education (t(30) = 1.965, p = .06), or on their performance on the fluid reasoning subtest (t(30) = 2.32, p = .43)”, previously L.161 to L255, in the beginning of the method.

2. ***I highly suggest to change “condition” for a meaningful term such a movements or anything that suits authors idea of what they are measuring through those three conditions. This will improve readers’ experience.***

**Response:** *We thank the reviewer for the suggestion and have adjusted the term “condition” by “point-light condition” in all the manuscript. The new terminology has been inspired by, previous studies such as. Campbell et al., 2011.*

***3. In results given within methods section, mean +- SD was not reported. Please complete the statistical reports.***

**Response:** Thank you for your observation. We added all the mean+/- SD missing in the first draft of the manuscript.

***4. Authors indicates that they used “global mean response”. I did not understand properly this normalization with the information given in the text. I search information about it, and I find many different conceptualizations for the same concept. As such, I would highly appreciate to include a detailed explanation of what was made in order to increase replicability of this work. Particularly the weighting method is not mentioned at all. Also, no cite for this method, or rationale behind it is given. As it is currently given, it is not easy to understand the properties of this normalization or the reasons to use it.***

**Response:** As explained in the manuscript (starting L.198), we used normalized reaction times because 1) we found a significant difference between the time duration of the three types of stimuli, 2) despite the instruction to press as fast and accurately as possible, all the participant (deaf and hearing) waiting for the end of the stimulus to answer. Therefore, we normalized reaction times based on a calculation below: each individual's response time to a video has been divided by the duration of that specific video then multiplied by the global mean, which represents the mean duration of all 126 videos combined (L212).

***5. Also, it is not clear when this normalization is used, as in Figure 1 Reaction times are plotted raw.***

**Response**: As explained above, we chose to normalize reaction times because the duration of the videos varied from two to four seconds in each point-light condition. Thus, Figure 1 represents normalized reaction times and all following statistical tests on the reaction time measure have been performed with these. We are confident that they represent an optimal measure of behavior during the recognition of biological motion.

***6. It is not justified why using the differences in reaction time (unclear if normalized or not) as covariates. I also did not understand in which statistical model they were introduce as covariates (which was the dependent variable). Since it is mentioned as start of new paragraph, I don’t understand if it refers to previous or following analyses. I assume, it refers to repeated measures ANOVA; however, this cannot be assessed with ANCOVA, as there is not properly developed repeated measures ANCOVA. This is usually assessed using Mixed Linear Models. In any case, there is no justification to include it in any of them as covariate. I would appreciate a clarification of this point.***

**Response:** The difference in reaction times for emblems and pantomimes is not normalized because reaction times were already normalized to total mean response time for the three conditions and video duration. There is also a significant difference in response times for both groups between conditions (behavioral differences statistical analysis). This difference accounts for the additional semantic processing time of emblems compared to pantomimes. The difference in RTs is then introduced in the 2^nd^ level (fMRI statistical analysis in SPM) regression model as a covariate to test correlation between reaction time change and brain signal change in Emblem-pantomime contrast across subjects.

***7. In line 244 authors say that they will use a correlation (without describing which method specifically) using an F-test. From results I induce that the authors used Pearson correlation, which used r-statistic instead of F statistic. F-statistic is used to evaluate the significance of a linear model, using t-statistic for each coefficient. Please clarify what are the authors exactly applying.***

**Response:** In this case, the 2^nd^ level analysis in SPM was applied using a multiple regression model introducing signal change for emblem-pantomime contrast as the dependent variable and the difference in reaction time between emblem and pantomime as a covariate for both groups, to test for covariation (either positive or negative) between behavioral performance and brain signal change.

The *t* statistic is used to test for Emblem-Pantomime contrast and the covariance with RT difference. As such for voxels which pass correction the signal change and performance are significantly correlated. The results in Figure 4 graph represent the relationship between behavioral change and brain activity at the voxel of maximum activity which value was extracted from SPM.

***8. In line 261-2 authors cite Figure 1E, but it only has until D.***

**Response**: We thank the reviewer for the suggestion and have corrected this error and replaced “Figure 1E” by “Figure 1D”.

***9. Figure 1 Caption appears 2 times in text body.***

**Response**: Thank you for your comment. We remove one on the legend under the name of “Figure 1” in the main text.

***10. In Table 2, authors wrote “most significant”. This is vague. Please explain the criteria by which those results were included and why some others were excluded of the report.***

**Response:** Thank you for your comment. Indeed, we re-phrased this sentence, instead of “MNI coordinates (x, y, z) of the most significant cluster are given (…)”,

We suggest: “MNI coordinates (x, y, z) of the significant cluster are given (…).” Thus, we applied this correction to all the MRI legends.

***11. In line 350, authors wrote “STG significantly predicted response time” base on (what I induce) a correlation. This is not appropriate. Correlations do not allow to talk about predictions or estimations, as coefficients are not estimated. Strictly speaking in this study only estimations can be made as there is no train and new data logic. I don’t mind the misusage of prediction as it may contribute to improves readers’ understanding of the work (most people in biological areas misused the concept), but then coefficients must be reported.***

**Response:** We thank the reviewer for the suggestion. We have strictly pursued statistical guidelines for correlations based on Cohen, J. 1988. Statistical Power Analysis for the Behavioral Sciences, 2nd Edition. Routledge. Thus, L. 372, we have reported all classical values: *p*, *r* and *R^2^* as coefficient. We re-phrased this sentence, instead of “STG significantly predicted response time”.

We suggest: “STG could predict response time”.

***12. In Figure 4 please include a color legend to improve reader’s experience.***

**Response:** Thank you, we agree with your suggestion. We have proceeded to minor changes in the Fig.4 to include a color legend. We believe this has helped to clarify the figure.

***13. Authors should consider the usage of F-test in correlations presented in Figure 4, considering that here authors imply a hypothesis that neural activity produce behavior (which makes total sense). In that case, it is better suited a linear model. Also, and considering the plots, particularly for left hemisphere, I would advise authors to fit a linear model and to remove outliers base on bonferroni’s outlier test (is model based, so authors must fit first the model, remove outliers and fit again). Author may also try to fit a model in y=b0+b (Reaction Time)+b(Reaction Time)squared. This would allow to capture the parabolic like pattern that this curve has in left hemisphere in deaf group. It is likely that increase in activity plateaus at some point, which would be consistent with non-linear modeling. This similar pattern seems to occur in NH for right STG. I would also encourage to include other relevant variables in the model which may contribute to reduce the noise. Personally, I think that the method used is not taking the best of the results obtained.***

**Response:** As explained above the statistical analysis for covariance between RT difference and brain activity was done in SPM as part of the imaging data statistical analysis. The use of a linear model is interesting but as long as there is a collinearity between brain activation and behavioral performance other variables are introduced instead of STG brain activation. The communication means (sign, aural) seems to predict well the change in RT. With signers showing reduced change in RTs between Emblems and pantomimes. See results:

***14. Finally, squared Rs derived from correlations are too weak to establish conclusive results. As such, depending on how linear modeling goes, authors may use bootstrap or any cross-validation technique to evaluate if fit is low but consistent. Otherwise, I would expect to moderate discussion considering that this result may not be obtained if the study is replicated.*
Response:** We will consider discussing this point in the paper, see L. 440-447.

**Report of Reviewer #2**

The authors study behavioral and fMRI measures of emblems and pantomimes gestures recognition, between early-deaf and hearing individuals. The total number of participants is 32 (16 early-deaf and 16 hearing subjects matched on age, sex and number of education years).
The article is very clear and well written. The methodology is sound, with stimuli that were carefully prepared, and the results were appropriately analyzed. Results are well presented and very interesting.

A main finding is the greater sensitivity to the processing of human action in early-deaf individuals (with a processing of emblems as fast as pantomimes) that is better explained by an effect of auditory deprivation. These behavioral differences where directly correlated with a bilateral activation of the STG. Also, a correlation was found between these activations and response time, suggesting that the extent of STG recruitment in deaf subjects depends on the capacity that have to detect emblems more rapidly than pantomime.

***My only concern is the size of the sample. If the paper is accepted, the authors should discuss the possible effects of using such a small sample... for example, there were no differences for the primary language of the deaf individuals (sign, native sign and spoken)... could this be due to the use of too small a sample?***

**Response:** Thank you for your comment, we agree that this information should be mentioned as one of the conclusions and have modified the manuscript to account for your comment. To express this, we suggest adjusting the wording with the following: “*In future studies, a larger sample size of deaf individuals would be needed since deafness related factors influencing brain plasticity (e.g. deafness duration, amount of residual hearing, prior use of hearing aids) and should be considered in the analyses (13,54).*” However, we are very confident with our result showing that behavioral difference was correlated to a bilateral activation in the STG in all our early deaf participants, independently of the primary mean of communication.

Finally, we want to report to the reviewer 2 the challenges that are linked to the recruitment of deaf individuals, especially for imaging research who are, at some point, disconnected to their daily-life preoccupation. Therefore, the specific characteristics of this population make it difficult to access through the usual recruitment “channels” despite support, in our case, from a major deaf rehabilitation center in Montreal. In previous studies, the number of deaf adults tested in imaging varied between 6 to 53 (see for example, Meyer et al., 2007 or Shibata 2007, Lepore et al., 2010 or Smithenaar et al., 2016). We recently performed a power analysis to estimate the optimal sample. The analysis suggests that a sample of 25 deaf participants is required to perform linear regression linked to deafness heterogeneity, thus, we hope that futures studies will replicate our main results while enriching them with regression analyzes.
