# Supplementary material for: Enhancement of visual biological motion recognition in early-deaf adults: Functional and behavioral correlates
Source: PLoS One. 2020 Aug 10;15(8):e0236800. doi: 10.1371/journal.pone.0236800 (PMC7416928; doi:10.1371/journal.pone.0236800)
Supplement: Attachment — Submitted filename: Response to Reviewers.docx [file pone.0236800.s002.docx]

We are happy to enclose a second revised version of the manuscript PONE-D-19-34925 entitled “Enhancement of Visual Biological Motion Recognition in Early-Deaf Adults: Functional and Behavioral Correlates” for consideration in *Plos One*.

We have carefully taken all the reviewer 1 suggestions into consideration to prepare a revised version of the manuscript, which also required modifications in the text and a revised figure. We truly feel the reviewer have contributed to improving the quality and clarity of the manuscript.

Below, you can find our responses to the comments of Reviewer 1 as well as actions taken in the revised manuscript.

We hope that you will find our revised manuscript satisfactory for publication and would be very proud to be given the opportunity to represent your journal.

**Report of Reviewer #1**

**Reviewer #1: Authors have addressed most of the comments given. However, there are still one major confusion which limits strongly the replicability of the reported results, and two minor comments about the Figures.

1- In line 251 to 254, the GLM method is briefly explained. First, there is no mention to the specific GLM used. From comments of the authors, I must assume that a Generalized Mixed Linear Model was used. Second, authors mention correlations (without mention which kind) analysis as synonym of GLM, which is not the case. Third, there is no mention to the structure of the GLMM used. Which variables were of first level, second level, were interaction specified? which ones, and so on. This is relevant, as introducing random effects and cross-level interactions are likely to produce overfit. Fourth, authors report Pearson r instead of beta coefficient, while saying that they used GLM. GLM uses F-statistic, not r-statistic. One can obtain r.-statistic from GLM; however, is not a proper results report and omit the most relevant information, like the beta value.
Since authors have been so consistent in using as synonym correlations, GLM and GLMM, I reviewed in detail the SPM documentation and documentation used for tutorial and divulgation purposes. In many of these documents it is stated that GLM implemented in SPM can be used to asses t-test, correlations, linear modeling, and generalized linear models. Correlations and t-test are not GLM; however, one can used particular cases of GLM to answer almost the same questions answered by t-test and correlations. In the case of ANOVA and ANCOVA, they are fairly the same test. However, in the case of correlation and t-test they are not equivalent to GLM. Discrepancies will respond mostly to assumption violation and sample size used. Suggesting them as synonym only contributes to obscure the methods used and limiting replicability.

I understand the source of the confusion. However, current statistical report limits strongly replicability and metanalytic work. To solve the current confusion, please:

a-Refer always to GLM always instead of correlation.**

**Response**: Thank you for your pertinent comment, this is now done when it applies.

**b-In method sections specify properly the structure of the GLM model used. Which variables in 1st and 2nd level, if any interaction was introduced, and which variables were introduced as fixed or random effects.**

**Response**: Thank you, we agree with your suggestion. We have proceeded to minor changes This section is now part of the manuscript:

*The General Linear Model for first level analysis predicted the bold response as the dependent variable and conditions: emblem, pantomime and scrambled point light movements as predictor factors of change in bold response.*

*The resulting individual contrasts (comparing estimated Betas to 0 using one tailed t-test) for each condition were smoothed and entered for the second level analysis.*

*For the second level analysis we used a full factorial design to estimate the effect of point light stimulation conditions between groups. The within subject factor is condition: Emblem, Pantomime, Scrambled. The between subject factor is group: Deaf vs controls.*

*Model estimates resulted in contrasts for the main effect of conditions, the main effect of group and the interactions between groups and conditions.*

*We also tested difference contrasts to assess specific directions of change using t-*tests*. Biological motion contrast was calculated as scrambled subtracted from the sum (Emblems + Pantomimes) in each group separately.*

*We contrasted biological motion, emblems and pantomimes between groups to assess specific activations in the deaf group for the processing of both types of gestures.*

*To assess the relation between change in brain activity and behavioral measures, we used a full factorial design group (2) by condition (3) and difference in response times as a covariate in the model.*

**c-Please check the SPM output if beta values (also referred as coefficients) are reported. It may be the case that if you are using random effects, these will not be reported, or they would not be of easy access. In that case, please remove the r-statistics of the current results report (p-values are not estimated based on such r-statistic, therefor is misleading to report it).**

**Response**: Once the model of covariation between changes in brain activity between groups and conditions and behavioral measures was estimated, areas showing significant covaration with performance measures were identified in bilateral STG regions.

Resulting brain activity time series in bilateral STG areas were calculated (at peak activity) and an independent correlation analysis was carried out using Pearson correlation coefficients to specify the relation between the behavioral measures (response times) and the cerebral activations in the left and right STG. Correlation results reported in the manuscript are related to this analysis.

This is now precised in the manuscript by adding a section.

**d-If possible, report the F-statistic from which GLM model significance is estimated (current draft does not report GLM significance, I recommend including it if possible). Also, if possible, include the t statistic usually used for GLMM coefficients.**

**Response**:

*F statistic for the main effect of group: F=22.5960 (p=0.05 FDR corrected)*

*F statistic for the main effect of condition: F=13.6698 (p=0.05 FDR corrected)*

*F statistic for the covariance with behavioral performance F=35.3696 (p=0.05 FDR corrected)*

**Minor**
**2- In Figure 3 B, C, D and in Figure 4, there is a yellow to white color scale without units. There is no explanation either of what these scales represent. Please include the variable to which is referring and the units of such variables***.*

**Response**: Thank you for your observation, we added the missing information.

*Figure 3: color scale represents T values*

*Figure 4: color scale represents F values*

**3- Figure 1 D, standard unit for milliseconds is ms, not MSEC. Capital M states Mega, which means *10^6, while m means *10^-3. Sec is an informal abbreviation; s is the standard unit for seconds.**

**Response:** We thank the reviewer for the suggestion and have corrected this error in the Figure 1D.
